# Up-regulation of MELK by E2F1 promotes the proliferation in cervical cancer cells

**DOI:** 10.7150/ijbs.62517

**Published:** 2021-09-07

**Authors:** Hongzhi Sun, Hongmei Ma, Hao Zhang, Minjun Ji

**Affiliations:** 1 Department of Orthopaedics, Nanjing Jiangbei Hospital affiliated to Nantong University, Nanjing, Jiangsu, 210048, China.; 2 Department of Obstetrics and Gynecology, Ma'anshan People's Hospital, Ma'anshan, Anhui, 243000, China.; 3 State Key Laboratory of Reproductive Medicine, Department of Histology and Embryology, Nanjing Medical University, Nanjing, Jiangsu, 211166, China; 4 Department of Pathogen Biology, Nanjing Medical University, Nanjing, Jiangsu, 211166, China.; 5 Jiangsu Province Key Laboratory of Modern Pathogen Biology, Nanjing, Jiangsu, 211166, China.

**Keywords:** MELK, HPV E6/E7, E2F1, MELK-8A, Cell arrest, Polyploidy

## Abstract

Cervical cancer is a common gynecologic cancer and a frequent cause of death. In this study, we investigated the role of MELK (maternal embryonic leucine zipper kinase) in cervical cancer. We found that HPV 18 E6/E7 promoted MELK expression by activating E2F1. MELK knockdown blocked cancer cells growth. Furthermore, we used MELK-8A to inhibit the kinase activity of MELK and caused the G2/M phase arrest of cancer cells. Under the treatment of inhibitors, Hela cells formed multipolar spindles and eventually underwent apoptosis. We also found that MELK is involved in protein translation and folding during cell division through the MELK interactome and the temporal proteomic analysis under inhibition with MELK-8A. Altogether, these results suggest that MELK may play a vital role in cancer cell proliferation and indicate a potential therapeutic target for cervical cancer.

## Introduction

Cervical cancer is a common gynecologic cancer. According to the latest global cancer statistics, the incidence and mortality of cervical cancer rank fourth among female malignant tumors, which cause serious harm to women's physical and mental health 1. Studies have shown that persistent infections with human papillomavirus (HPV) are high-risk factors for cervical cancer in early marriage and early childhood 2. At present, there have been many reports on the relationship between human HPV infection and the development of cervical cancer. HPV is a closed circular double-stranded DNA virus. The viral gene component is divided into three parts: the early gene region (E), the late gene region (L), and the long regulatory region (LCR) that separates the early region from the late phase. The regulatory region mainly regulates the transcription of viruses, controls the production of viral proteins and infectious particles. The early region encodes E6, E7, E1, E2, E4, and E5 proteins, which are mainly involved in the replication and transcription of viral DNA. The early expression genes E6 and E7 are oncogenes, and their encoding viral oncoproteins play a crucial role in cell transformation and maintenance of the malignant phenotype of transformed tissues. The HPV genome integrates into the human genome during the process of cancer development. The E6/E7 genes persist during the integration process, and the L1 gene may be lost 3. The E6/E7 proteins significantly mediate the degradation of the tumor suppressor genes p53 and Rb, thereby activates the transcription factor E2F1 and interferes with cell cycle regulation 4. The persistent infection of HPV 16/18 has caused about 70% of cervical cancer in the world. Breakthroughs in the immune system, activation of proto-oncogenes, inactivation of tumor suppressor genes, and overexpression or loss of multiple factors are involved in this process 2.

We focused on the study of the maternal embryonic leucine zipper kinase (MELK) by analyzing 32 samples (12 control samples, 20 cervical cancer samples) using the Illumina HumanHT-12 V4.0 expression bead chip microarray platform 5. MELK mRNA is more highly expressed in cervical cancer tissues than normal cervical tissue, and MELK expression increased in cervical cancers from grade 1 to grade 3. No matter in cervical adenocarcinoma or cervical squamous cell carcinoma, the expression of MELK is higher in cancer tissues than in adjacent tissues 6. The TCGA tumor database analysis found that the MELK RNA level in cervical cancer tissue was significantly higher than other tumor tissues. Based on the above data, we raised the questions of whether HPV is an important factor causing high expression of MELK in cervical cancer and whether the increased expression of MELK can change the biological characteristics of cervical cancer cells.

MELK structure is highly conserved. It consists of an N-terminal Ser/Thr kinase domain, a ubiquitination-related domain, and a regulatory region at the C-terminus, belonging to a cycle-dependent kinase 7. In 1997, it was first identified in a mouse cDNA library of egg cells and embryos. The gene was mapped to mouse chromosome 4 and named MPK38 in the mouse 8. Subsequently, in 2002, scientists discovered another Eg3 protein (pEg3) related to cell cycle regulation in toads. Studies have confirmed that human MELK, rat MPK38, and toad pEg3 are highly homologous 9. MELK belongs to the Snfl/AMPK kinase family. Unlike other members of the family, MELK is not involved in cell survival regulation under metabolic stress. It is mainly involved in apoptosis, cycle regulation, cell proliferation, tumorigenesis, embryonic stem cell turnover 10. MELK is highly expressed in cells and tissues that have a solid ability to divide and self-renew. In the currently confirmed studies, MELK expression is deficient in normal heart, liver, kidney, and other tissues or organs, but the expression is significantly elevated in a variety of human tumors such as melanoma 11, colorectal cancer 12, breast cancer 13, and glioblastoma 14.

Note that MELK could play an essential role in mitosis. It demonstrates increased protein abundance during mitosis and is degraded when cells progress into the G2 phase 15,16. It should be mentioned here that there is a lack of mechanistic understanding of the role of MELK during cell division. An immediate question is the identity of the MELK substrates that mediate its role in mitosis, such as promoting mitotic cell survival.

In the current study, we aimed to investigate the role of MELK in human cervical cancer cells. We found that HPV18 E6/E7 promoted the expression of MELK by activating E2F1. The deletion or inactivation of MELK leads to abnormal cell division and apoptosis in cancer cells. We also comprehensively characterized the MELK interactome and revealed insight into critical cell biological processes after inhibition with MELK-8A through temporal proteomic profiling. Our research reveals that MELK plays a vital role in the proliferation of cervical cancer cells and provides new targets for developing drugs and diagnostic markers for the treatment of cervical cancer.

## Materials and Methods

### Cell culture

HeLa, SiHa, CaSki, and C33A were bought from ATCC (American Type Culture Collection) (Manassas, VA, USA). HeLa and SiHa cells were cultured in DMEM media (WISENT). CaSki and C33A were cultured in RPMI 1640 media (WISENT). They were supplemented with 10% fetal bovine serum (Invitrogen) and 0.5% Penicillin-Streptomycin Solution (Gibco, Waltham, MA, USA), maintained at 37 °C in humidified air with 5% CO2.

### Sample collection and immunohistochemistry staining (IHC)

The immunohistochemistry procedures were as described previously 17. The study included 12 patients with cervical cancer diagnosed at the Department of Oncology-Pathology, second people's Hospital, Nanjing, China. The study was performed with the approval of the Ethics Committee of second people's Hospital. All the fresh samples were immediately kept in liquid nitrogen, and the clinical characteristics were recorded. The sections were subjected to dewaxing in xylene and dehydration in graded ethanol. The activity of endogenous peroxidase was blocked by 3% H_2_O_2_ for 10 min. The sections were then heated to 100 °C in 0.1 M citrate buffer (pH 6.0) for half an hour to retrieve the antigens. These tissues were incubated with anti-MELK overnight at 4 °C. After washing with phosphate-buffered saline (PBS), tissues were incubated with the second antibody, which was horseradish peroxidase-conjugated anti-rabbit IgG (Envision kit, Dako, Denmark), following the manufacturer's instructions. At last, sections were stained with hematoxylin. These stained tissues were photographed with a microscope from Carl Zeiss (Axio Observer A1, Jena, Germany). As described previously 18, the staining results were scored by two investigators blinded to the clinical data. Then the expression of MELK in cervical cancer was analyzed by the Chi-square test. Positive reactions were defined as those showing brown signals in the cell cytoplasm. A staining index (values 0‐12) was determined by multiplying the staining intensity score with the positive area score. The intensity was scored as follows: 0, negative; 1, weak; 2, moderate; and 3, strong. The frequency of positive cells was defined as follows: 0, <5%; 1, 5%‐25%; 2, 26%‐50%; 3, 51%‐75%; and 4, more than 75%. For statistical analysis, scores of 0‐7 were considered the low expression, and scores of 8‐12 considered high expression.

### Cell line construction

Plasmids of shRNAs targeting HPV18 E6/E7, MELK, and scramble were purchased from GenePharma (Shanghai, China). siRNA targeting E2F1 and scramble were synthesized by GenePharma (Shanghai, China). The shRNA of MELK or scrambled was transfected into HeLa or CaSki cells using lipofectamine 2000 (Invitrogen, California, USA), followed by 40 days of selection with 800 μg/ml of puromycin. All genes knockdown in these cells were confirmed by Western blot. Other plasmids are transiently transfected using lipofectamine 2000.

### Immunoblotting analysis

Cells were lysed with Triton-x 100 buffer (Beyotime P0013) containing protease inhibitor cocktail and phosphatase inhibitor cocktail (Beyotime). The proteins were separated by electrophoresis using 8% or 12% ExpressPlusTM SurePage gel from GenScript (Nanjing, China) and transferred to the nitrocellulose membrane. After blocking, the membrane was then incubated overnight with primary antibodies. After washing in TBST, secondary antibodies were incubated for about 2h depending on the primary antibodies, washed three times, and then detected.

### Xenograft tumor growth assay

All the animal experiments were approved by the Institutional Animal Care and Use Committee of Nanjing Medical University (SYXK 2015-0015). All animals received human care, and all animal experiments were conducted following the relevant guidelines and regulations. The animals were bought from Beijing Vital River Laboratory Animal Technology Co., Ltd. (Beijing, China). About 6×10^6^ HeLa cells (200 μL) stably transfected with shNC or shMELK were injected subcutaneously into the right flank of each athymic mouse (4 weeks old female nude mice, n = 4). Mice were housed in cages with sawdust bedding in a specific pathogen-free room at a temperature of 25-26 °C and relative humidity of ~50%, light 12 h/day. Tumor size was measured by calipers every three days. Tumor volume was assessed, conforming to the formula: volume = (longest diameter × shortest diameter^2^)/2^19^. Mice were sacrificed on the fortieth day after the beginning injection of HeLa cells. Then, the tumor tissues were harvested for further studies.

### Cell proliferation assay and colony formation assay

After planting 5,000 cells in a 96-well plate for 12 hours in adherent culture, a kinase inhibitor MELK-8A was added to the plate for 24, 48, and 72 hours. At last, cell proliferation was detected using CCK8 reagent (YEASEN; China). During the assay to examine colony formation, about 2,000 cells per dish were plated in a 6-well plate. After about 6 hours, the cells were adherent, and the original medium was replaced with a 10% complete medium with the appropriate inhibitor concentration. After seven days, the cells were fixed with 4% paraformaldehyde for 30 min and stained with 0.5% crystal violet for 30 minutes, then gently washed with PBS three times. The number of cell colonies was calculated.

### Cell cycle analysis

For the BrdU incorporation assay, cells were cultured with DMSO control or inhibitor for 24 hours, and the cell cycle was analyzed by BD Pharmingen FITC BrdU Flow kit (Becton Dickinson, San Jose, CA) according to the manufacturer's instructions. The fluorescence signal was quantified by flow cytometry (FACS LSRII; Becton Dickinson) using Flow Jo software (Treestar, Ashland, OR). After adding 40% of the digested cells to the 6-well plate and incubating the cells for 12 hours overnight, the kinase inhibitor was added and cultured for 24 hours. Finally, cells were collected by trypsinization after the PBS washing, fixed in 70% cold ethanol, and followed by treatment with RNase and propidium iodide in PBS for the FACS analysis.

### Immunoprecipitation

Cell lysis buffer for Western and IP (P0013; Beyotime), Protease and phosphatase inhibitor cocktail for general use, 50X (P1045; Beyotime). Cleared lysates were incubated with anti-human IgG magnetic beads to eliminate proteins with nonspecific binding to immunoglobins and beads. The lysates were incubated overnight with Anti-DYKDDDDK-Tag Human mAb (Agarose Conjugated) (M2008; Abmart) at 4 °C. The beads were washed with lysis buffer five times before SDS sample buffer was added to prepare samples for immunoblotting. For mass spectrometry assay, proteins binding to anti-Flag beads were eluted using Flag peptides (F4799; Sigma) and precipitated by trichloroacetic acid. The sample was submitted to the AIMS Scientific Co., Ltd. (Shanghai, China) for mass spectrometry analysis.

### LC-MS/MS Analysis

The peptide samples were analyzed on Thermo Fisher LTQ Orbitrap ETD mass spectrometry. Briefly, loaded sample onto an HPLC chromatography system named Thermo Fisher Easy-nLC 1000 equipped with a C18 column (1.8 mm, 0.15×1,00mm). Solvent A contained 0.1% formic acid, and solvent B contained 100% acetonitrile. The elution gradient was from 4% to 18% in 182 min, 18% to 90% in 13 min solvent B at a flow rate of 300mL/min. Mass spectrometry analysis was carried out at the AIMS Scientific Co., Ltd. (Shanghai, China) in the positive-ion mode with an automated data-dependent MS/MS analysis with full scans (350-1600 m/z) acquired using FTMS at a mass resolution of 30,000 and the ten most intense precursor ions were selected for MS/MS. The MS/MS was acquired using higher-energy collision dissociation at 35% collision energy at a mass resolution of 15,000.

### Identification and quantification of Proteins

Raw files were searched against the human protein sequences obtained from the Universal Protein Resource (UniProt) database using Maxquant software. False discovery rates (FDR) were estimated using the target-decoy strategy, and FDR cut-offs were set to 0.01 for peptides and proteins. Enzyme specificity was considered full cleavage by trypsin, and two maximum missed cleavage sites were permitted. The minimum required peptide length was set to 6 residues. Carbamidomethyl (C) was set as fixed modification. Variable modifications included oxidation (M) and acetylation (protein N-term). The mass tolerance for precursor ions was set to 20 ppm at the first search as applied in Maxquant for initial mass recalibration. For the main search, the mass tolerance for precursor ions was set to 6 ppm. The mass tolerance for fragment ions was set to 0.5 Da. Quantification was via MaxQuant's LFQ algorithm, which combines and adjusts peptide intensities into a protein intensity value. For the MELK interactome, interactors with high intensities in the pulldown group (top 10%) were considered high confidence. The criteria for differentially expressed proteins in the temporal proteomic analysis was an 8-fold change between maximal and minimal value within DMSO, 24 h and 48 h groups.

### Bioinformatic analysis

Gene ontology (GO) and pathway enrichment analysis were performed using the function enrichGO of the clusterProfiler package 20 in the R programming environment. The resulting GO terms were analyzed for semantic similarity (cut-off value of 0.7) with GOSemSim 21 R package to reduce redundancy. Selections of non-redundant enriched terms (p-value<0.05) from the GO analysis were plotted. Visualization of results was performed using the ggplot2 R package 22. Heatmap for the temporal proteomic analysis was plotted using the ComplexHeatmap R package 23 following the k-means cluster.

### Additional methods

Details of reagents used for *in vivo* studies and cell lines, culture conditions, transfection, Western blot, immunofluorescence, immunohistochemistry, antibodies, primers, probes, and oligonucleotides are described in Supplementary Table S1.

## Results

### MELK is overexpressed in cervical cancer

**Figure 1:**
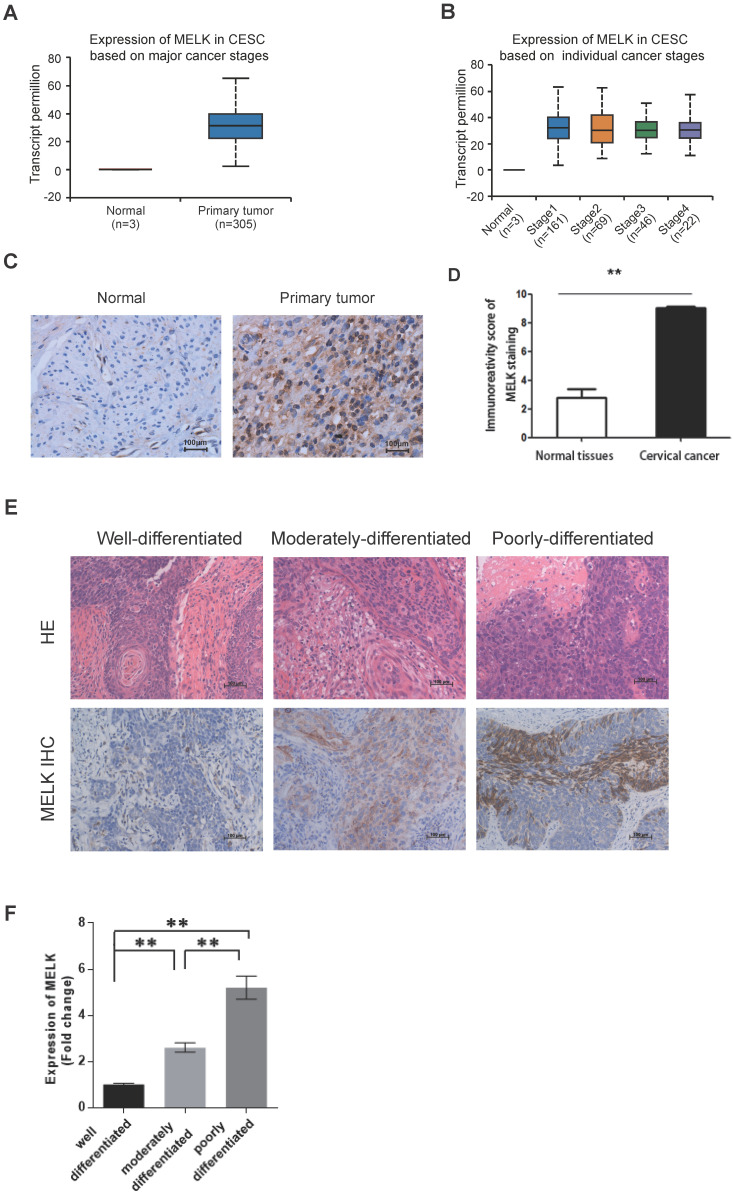
** MELK is overexpressed in cervical cancer. A.** The expression of MELK in human normal cervical tissues and cervical cancer tissues from the TCGA-CESE project. **B.** The expression of MELK in different cancer stages from the TCGA-CESE project. **C.** Immunohistochemical staining for the expression of MELK in adjacent normal human cervical tissue and cervical cancer tissue. Scale bars correspond to 100 µm. **D.** Quantification of immunohistochemical staining for MELK. A Chi-square test was performed to compare samples. * P <0.05, ** P <0.01, and *** P <0.001. **E.** Representative images of immunohistochemical staining for MELK in Well-differentiated (n = 4), Moderately-differentiated (n = 4), and Poorly-differentiated (n = 4) cervical cancer tissue. Scale bars correspond to 100 µm. **F.** Quantification of immunohistochemical staining for MELK in different differentiation levels of cervical cancer. A Chi-square test was performed to compare samples. * P <0.05, **P<0.01, and ***P<0.001.

To define that MELK plays a vital role in the progression of human cervical cancer, we first analyzed the expression of MELK in previously published gene expression datasets of patient-derived cervical cancer samples. MELK was overexpressed in primary tumors compared to normal samples (Figure 1A). In addition, MELK overexpression showed little difference in cervical cancer tissues, no matter in different cancer stages (Figure 1B), or patients' race, weight, and age (Figure S1A-C).

We also collected cervical tissues and detected MELK expression in paraffin sections of cervical cancer samples and their adjacent normal samples. According to the TCGA data, we verified by immunohistochemistry that the expression of MELK in cervical cancer tissues was indeed higher than that in adjacent tissues (Figure 1C,D). Furthermore, MELK showed much more positive staining in poorly differentiated tumors than in well-differentiated tumors and adjacent normal cervical tissues (Figure 1E,F). It suggested that MELK expression was positively associated with tumor occurrence.

### HPV18 E6/E7 protein promote MELK through regulating transcription factor E2F1

**Figure 2:**
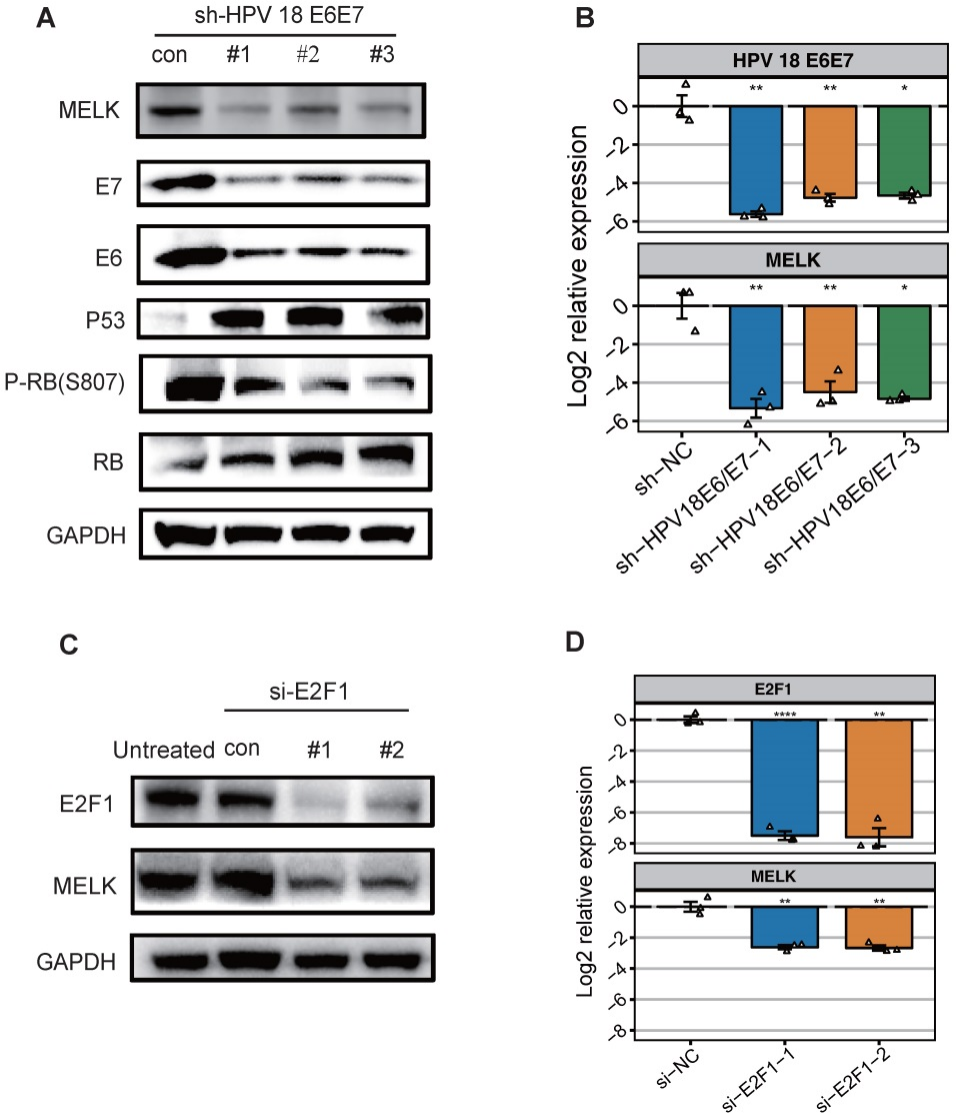
** HPV 18 E6/E7 proteins promote cervical cancer proliferation by up-regulating MELK through regulating transcription factor E2F1. A.** Effects of HPV18 E6/E7 knockdown on MELK expression. HeLa cells were treated with HPV18 E6/E7 specific shRNA for 48h, and levels of total or phosphorylated proteins were determined by Western blot. GAPDH was used as a loading control. **B.** mRNA expression for the indicated genes (MELK, HPV 18 E6, HPV 18 E7) was measured in HeLa cells 48 hr after shNC, HPV18 E6/E7 specific shRNA. * P <0.05, ** P <0.01, *** P <0.001 and **** P <0.0001 compared to the shNC group. **C.** HeLa cells were treated with E2F1 specific siRNA for 72h, and levels of total proteins were determined by Western blot. GAPDH was used as a loading control. **D.** mRNA expression for the indicated genes (MELK, E2F1) was measured in HeLa cells 72h after siNC, E2F1 specific siRNA. * P <0.05, ** P <0.01, *** P <0.001 and **** P <0.0001 compared to siNC group.

It is commonly accepted that high-risk HPV E6 targets p53 for degradation, which in turn inhibits the p53/p21 (WAF/CIP) pathway and ultimately activates the cyclin/CDK complex, causing the RB protein to shift from a hypophosphorylated state to a hyperphosphorylated state, disassembling the E2F/RB complex and promoting the freeing of the E2F protein. In addition, E7 protein can compete with E2F to bind RB proteins and degrade them 24-27. Taken together, E6/E7 will eventually promote the freeing of E2F and activate the transcription of E2F-responsive genes. To decipher the mechanism of MELK overexpression in cervical cancer, we knocked down the expression of HPV18 E6/E7 in HeLa cell lines using short hairpin RNAs (shRNAs) and analyzed the effect of E6/E7 knockdown on MELK expression. We found that MELK protein and RNA levels were significantly reduced with the low expression of E6/E7. Besides MELK, the expression of other proteins, including the phosphorylation of RB (P-RB), was also decreased. P53 and RB proteins expression were increased, consistent with the reported results 4 (Figure 2A,B).

To further explore what factors can regulate MELK overexpression, we predict E2F1 as a MELK transcription factor (Figure S1D) by published CHIP-seq data. Therefore, we individually knocked down E2F1 expression in HeLa cell lines using small interfering RNAs (siRNAs) and analyzed the expression of MELK. Knockdown of the transcription factor E2F1 significantly attenuated MELK protein and RNA levels (Figures 2C,D). Altogether, these results demonstrated that E2F1 is most likely a transcription factor of MELK.

### MELK is essential for cervical cancer proliferation

**Figure 3:**
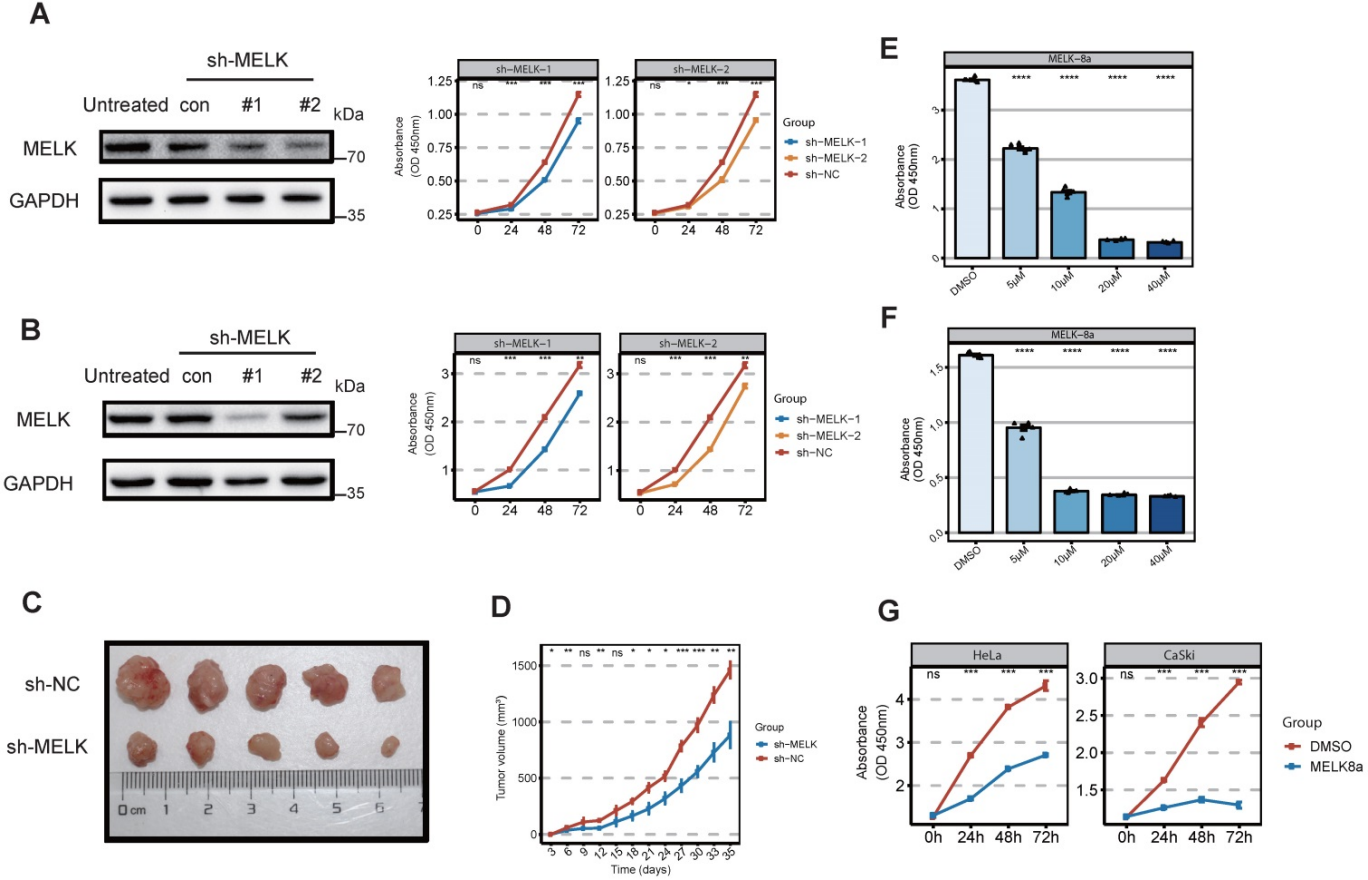
** MELK is essential for cervical cancer proliferation. A.** Effect of MELK knockdown on cell growth. HeLa cells are stably transduced with short hairpin MELK(sh-MELK). Then use the CCK8 proliferation assay kit to detect the proliferation of HeLa cells under different treatments. * *P* <0.05, ** P <0.01, *** P <0.001 and **** P <0.0001 compared to control. **B.** Effect of MELK knockdown on cell growth. CaSki cells are stably transduced with short hairpin MELK (sh-MELK). Then use the CCK8 proliferation assay kit to detect the proliferation of CaSki cells under different treatments. * P <0.05, ** P <0.01, *** P <0.001 and **** P <0.0001 compared to control. **C.** MELK-knockdown HeLa cells were injected into nude mice, and the tumors were harvested. **D.** The volume of tumors was subsequently measured every 3 days (mean ± SD); * P <0.05, ** P <0.01, *** P <0.001 and **** P <0.0001 compared to the shNC group. **E and F.** Viability of (G) HeLa and (H) CaSki cells 24 hours posttreatment with MELK-8A. Cell viability in a dose-dependent manner 24 hours posttreatment. DMSO was used as a control; * P <0.05, ** P <0.01, *** P <0.001 and **** P <0.0001 compared to the DMSO group. **G.** Effect of MELK kinase activity on cell proliferation after 5μM MELK-8A inhibition. The cell viability of HeLa and CaSki cells treated with MELK-8A was measured every 24 hours for 72 hours using the CCK8 cell proliferation assay kit. * P <0.05, ** P <0.01, *** P <0.001 and **** P <0.0001 compared to the 0h group.

Considering that MELK is highly expressed in cervical cancer, we would like to test whether MELK is a potential target for cervical cancer therapy. We treated cervical cancer cell lines with the short hairpin RNAs (shRNAs) against MELK to observe whether MELK knockdown would block cervical cancer growth. Our results showed that MELK knockdown significantly inhibited the proliferation of cervical cancer cells (HeLa and CaSki) (Figure 3A,B). *In vivo* experiments confirmed that knocking down MELK in HeLa cells significantly reduced tumor growth rate (Figure 3C,D). To determine whether the ability of MELK to promote cervical cancer growth was dependent on its kinase activity, we treated cells with MELK inhibitors (MELK-8A) 28. We used the different concentrations of MELK-8A in cervical cancer cells (HeLa, CaSki, and C33a) and found that the greater the inhibitor concentration, the lower the cell proliferation ability in a dose-dependent manner (Figure 3E,F and Figure S2A). We used MELK-8A on the different cervical cancer cell lines (HeLa, CaSki, SiHa, and C33a) and then tested the proliferation of cells for three consecutive days. As the time of MELK-8A treatment of tumor cells increased, the cell proliferation activity gradually decreased (Figure 3G and Figure S2B). These results suggested that when the MELK kinase activity was inhibited, the cell proliferation ability was significantly decreased, and apoptosis occurred after 48 hours.

### MELK is associated with mitosis in cervical cancer cells

**Figure 4:**
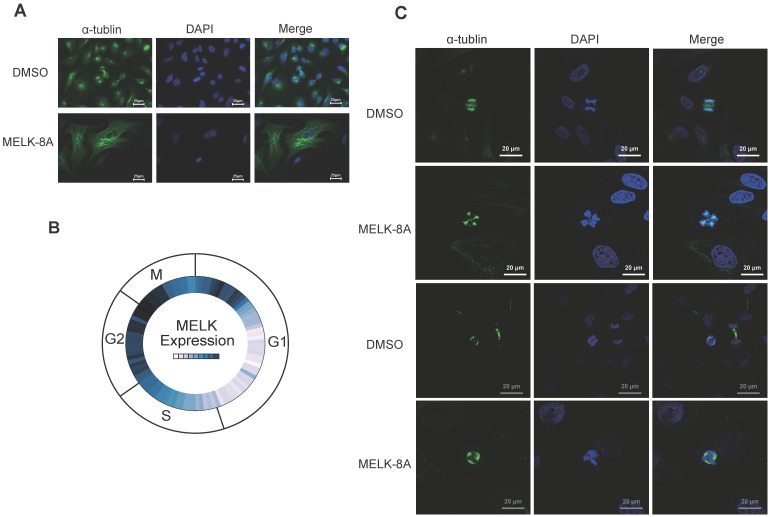
** MELK is associated with mitosis in cervical cancer cells. A.** HeLa cells were treated with DMSO or 5μM MELK-8A for 24 h. Mitotic spindles and DNA were visualized by immunofluorescent staining for α-tubulin and by staining with DAPI, respectively. DMSO was used as a control. Scale bars correspond to 25 µm. **B.** Expression of MELK in the cell G2/M phase from the Cyclebase3.0 cell cycle database. **C.** HeLa cells were treated with DMSO or 5 µM MELK-8A for 48h and 72h. Mitotic spindles and DNA were visualized by immunofluorescent staining for α-tubulin and by staining with DAPI, respectively. For MELK-8A-treated cells, normal mitotic phases were not found. DMSO was used as a control. Scale bars correspond to 20 µm.

To observe the changes in cell morphology with or without MELK-8A stimulation, we observed the internal structure, including cytoskeletal protein and nucleus of cervical cancer cells, by immunofluorescence technology. To our surprise, after Hela cells were treated with 5 μM MELK-8A for 24 hours, the cytoskeleton of the drug-treated group was significantly larger than that of the DMSO control group, and some cells had multiple nuclei phenotypes (Figure 4A). We further examined the expression of MELK in different cycles of the cell in the Cyclebase3.0 database and found that the RNA expression level of the MELK is mainly concentrated in the G2/M phase (Figure 4B). We continuously applied MELK-8A on HeLa cells, and internal changes were observed at 48 and 72 hours, respectively. The results showed multiple centrosomes inside the cells in the dividing phase, and the chromatin was disordered. The nucleus of MELK-8A-treated Hela cells was significantly larger than those of the control tumor cells (Figure 4C). It seems that MELK has a regulatory effect on the mitosis of tumor cells as polyploidy occurs in cervical tumor cells.

### MELK regulates multiple G2/M phase essential proteins and cell apoptosis

**Figure 5:**
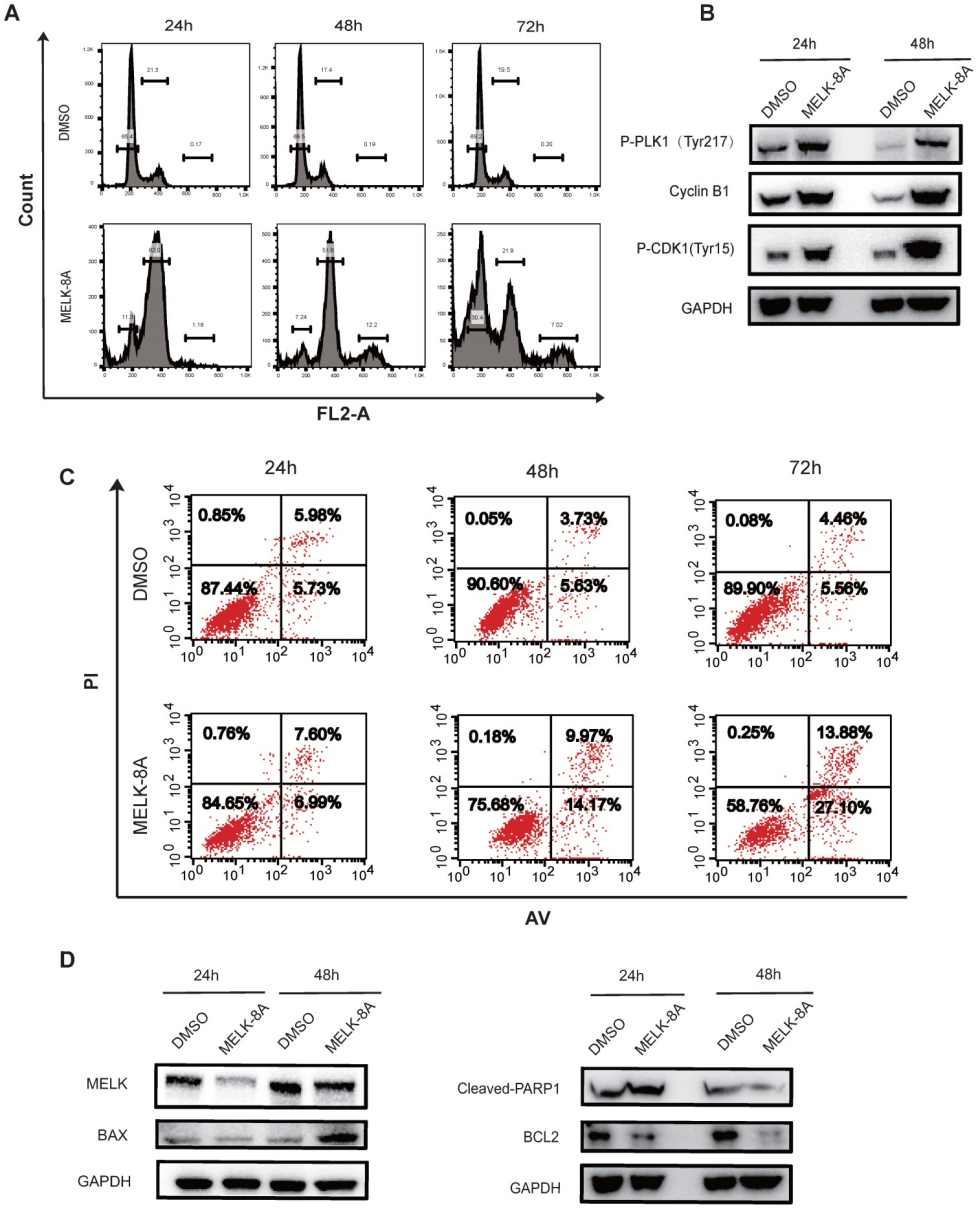
** MELK regulates multiple G2/M phase essential proteins. A and B.** After MELK kinase activity was inhibited by 5 µM MELK-8A, HeLa cells showed polyploidy. DMSO was used as a control. **C and D.** HeLa cells were treated with 5 µM MELK-8A for 48 h, and levels of total proteins were determined by Western blot. GAPDH was used as a loading control. Note that the protein abundance of P-PLK1^Tyr217^, Cyclin B1, P-CDK1^Tyr15^, BAX, and Cleaved-PARP1 increases, but MELK or Bcl-2 was reduced after use of MELK-8A.

Next, we adopted flow cytometry to detect cell cycle changes following inhibition of kinase activity. Hela cells were continuously treated with MELK-8A (5μM) for 72 hours, and a periodic change was detected every 24 hours. It was found that a large proportion of G2/M phase arrest occurred in Hela cells after MELK-8A was administered for 24 hours, and a significant proportion of polyploids occurred at 48 hours after MELK-8A administration. When MELK-8A continued to act for 72 hours, the ratio of the G1/S phase markedly increased, and polyploidy occurred compared to the groups with 24-hour and 48-hour drug treatment (Figure 5A). We also detected cell apoptosis by flow cytometry. The results showed that the early apoptosis and late apoptosis of the drug-treated group were significantly higher than those of the DMSO control group with the prolongation of the action of the small molecule inhibitor drug (Figure 5C).

To make sure what proteins were involved in the G2/M phase arrest when MELK kinase activity was inhibited, we examined changes in the expression of cell cycle and apoptosis-associated proteins. It was found that the levels of tyrosine phosphorylation at position 217 of PLK1, Tyr15 phosphorylation at CDK1, and expression of CyclinB1 were significantly increased after MELK-8A inhibited MELK kinase activity. Additionally, the apoptosis-related proteins were also affected along with MELK inhibition, including the increased expression of pro-apoptotic factor BAX, caspase 3-activated index protein cleaved-PARP1 proteins, and the decreased expression of anti-apoptotic factor BCL-2 protein (Figure 5D).

Taken together, we initially determined that inhibition of MELK kinase activity, MELK regulates multiple G2/M phase essential proteins and cell apoptosis.

### MELK-interacting proteins analysis by mass spectrometry

**Figure 6:**
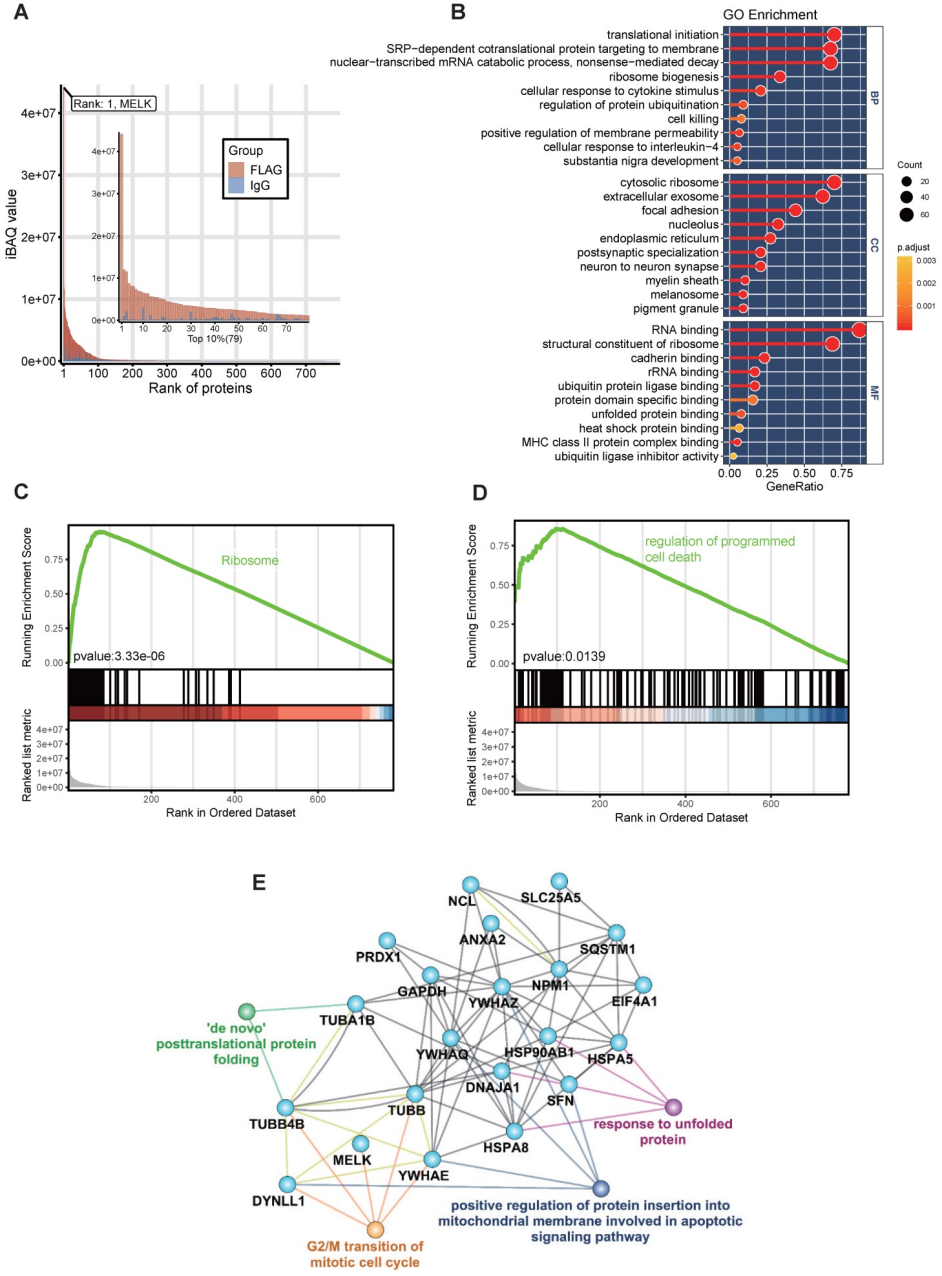
** Analysis of MELK protein interactome. A.** Bioinformatics analysis of 790 proteins in MELK interactome, focusing on the top 10% of the proteins. **B.** GO analysis of MELK interactome. **C and D.** Two enriched GSEA terms of the MELK interactome. **E.** A protein-protein interaction network based on MELK interactome and the related biological processes.

To reveal the role of MELK in the process of cervical cancer cell division, we tried to identify the proteins of co-localization and interaction with MELK protein and explore the potential biological functions of MELK. We constructed a FLAG-tagged MELK fusion plasmid, transient transfected it into Hela cells, co-immunoprecipitated, then analyzed by liquid-phase mass spectrometry (LC-MS/MS). By comparing the differences between the Anti-Flag group and the IgG group, we identified a total of 790 proteins that were enriched in the Anti-Flag group as MELK-interacting candidate proteins. In order to accurately describe the interaction of MELK, we selected the top 10% (79 proteins) of the candidate proteins as highly reliable interacting proteins or proteins that strongly interact with MELK (Figure 6A and Supplementary Table S2). The GO enrichment analysis found that these interaction proteins are mainly related to translation initiation, co-translational transport, ribosome components, RNA binding, etc. (Figure 6B).

In the GSEA analysis based on the KEGG pathway database, we found that the high signal intensity proteins in the MELK interaction protein profile are mainly ribosome-related genes, suggesting that MELK may be involved in mRNA translation during cell division (Figure 6C). In addition, genes involved in the regulation of programmed cell death pathways also exhibit higher signal intensities in the MELK interactome (Figure 6D). In order to reveal the relationship between MELK interaction proteins, we constructed an interaction network excluding ribosomal proteins using the GeneMANIA database 29 and Cytoscape software 30. The protein-protein interaction network has 21 protein nodes and 67 protein interactions. Because of the unknown protein interactions between some proteins, proteins without any interaction are not retained in the network. The annotation of GO information, the creation of nodes and connections for protein nodes in the network can lead to biological events in which interacting proteins may be involved and can also be presumed to be the biological functions of MELK proteins. The network analysis results showed that MELK, TUBB, TUBB4B, DYNLL1, and YWHAE were involved in the G2/M phase transition of cells, while DYNLL1, YWHAE, and YWHAQ, YWHAZ, and SFN were related to apoptosis signaling pathways. In addition, TUBA1B, TUBB4B, HSPA5, HSPA8, HSP90AB1, DNAJA1, etc., are all related to the post-translational folding of proteins (Figure 6E).

### Quantitative analysis of MELK-8a induced Hela proteome

**Figure 7:**
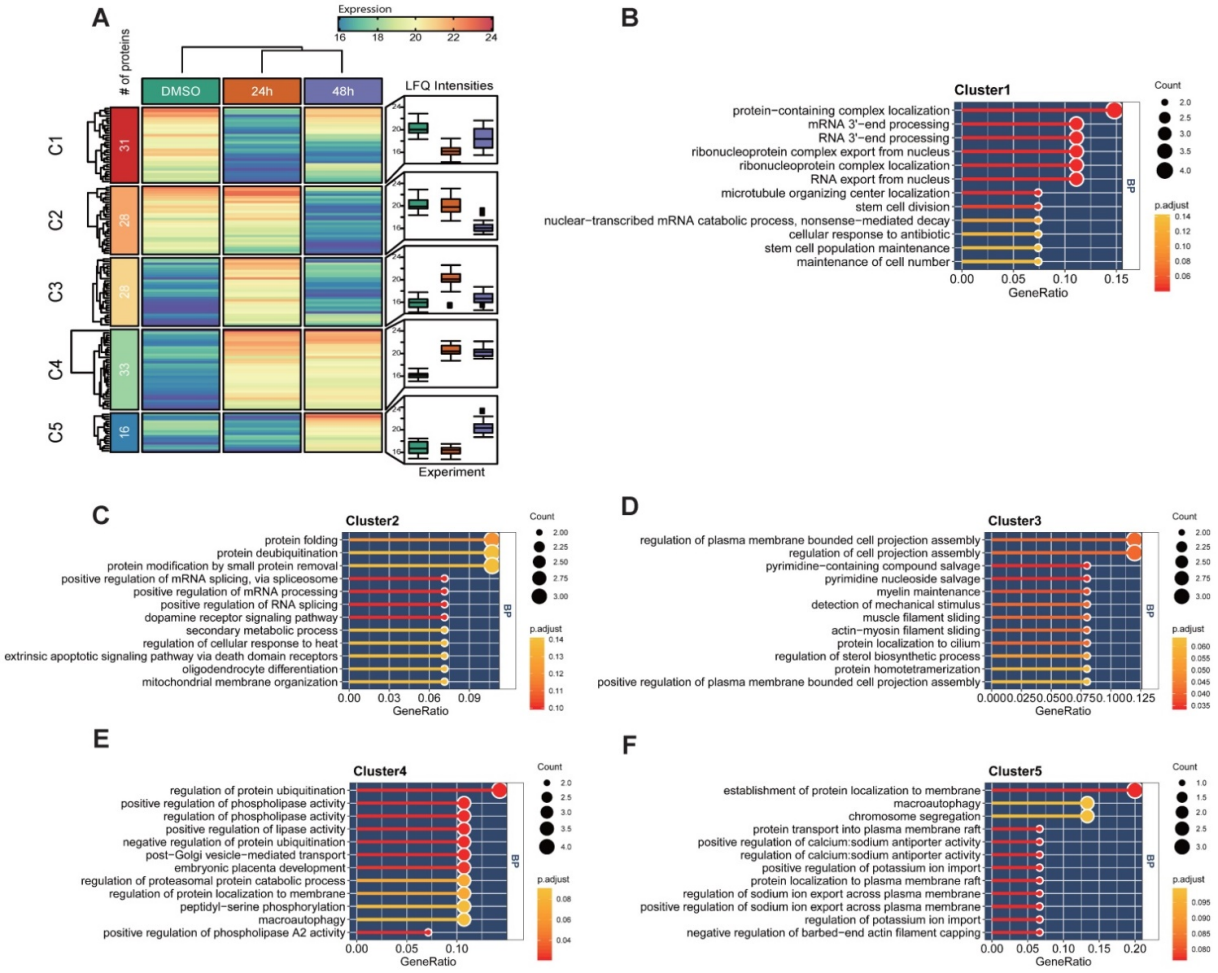
** Quantitative proteomic analysis of MELK-8a treated Hela cells. A.** Heatmap of differentially expressed proteins between three groups. Proteins were grouped into 5 clusters using the k-means algorithm. Box plot was used to visualize the distribution of LFQ intensities for each group. **B-F.** GO enrichment analysis for each group of differentially expressed proteins.

The previous experiment found that Hela cells were arrested in the cell cycle and morphologically changed under a microscope after MELK inhibition. We performed a temporal quantitative proteome on MELK-8A-treated Hela cells to investigate the proteins involved in this change. We set a strict 8-fold change cut-off for differentially expressed proteins due to the limit of our sample number and the label-free quantification method. In total, 136 proteins were grouped into five clusters using the k-means algorithm (Figure 7A and Supplementary Table S3). GO enrichment analysis indicated that proteins decreased in 24h (C1) after MELK-8A inhibitor treatment were mainly involved in RNA 3'-end processing (Figure 7B). Interestingly, these proteins were partially restored expression after one cell cycle. On the contrary, proteins related to protein-folding, deubiquitination, and phospholipase activity (C2-C4) were increased to or keep a high expression level after 24h (Figure 7C-E). Furthermore, 48h specifically expressed proteins (C5) were mainly involved in the establishment of protein localization to membrane, macroautophagy, and chromosome segregation (Figure 7F). These dynamic changes in protein expression may help to understand the biological mechanism for cell arrest and polyploidy.

## Discussion

Finding novel treatment opportunities for cervical cancer is a major task in current work. In this study, we show that MELK is important for cervical cancer growth. Our study allows us to draw several important conclusions. First, MELK expression was activated by the HPV E6/E7 via E2F1 and was necessary for cervical cancer growth. Second, we found that MELK is associated with several previously reported genes important for cell cycle progression, especially at the G2/M phase. Third, when the novel small molecule inhibitor MELK-8A acts on cervical cancer cells, we unexpectedly found that many sets of spindles appeared inside the surviving tumor cells, and the nuclei became larger than the control group, eventually leading to apoptosis. Finally, our proteomic analysis identified some interactors or the kinase substrates of MELK and constructed a protein-protein interaction network using the leading 79 interacting proteins. We found that MELK may be related to the translation of M-phase protein, mitochondrial membrane insertion, and apoptosis of cells, which provides a perspective suggestion for the mechanism of action of MELK in cervical cancer cell division. These results are significant because they describe the role of MELK in cervical cancer as a survival kinase. Our study also indicates that pharmacological inhibition of MELK with MELK inhibitor can exert strong inhibitory effects on tumor growth in various cervical cancer types, including cervical adenocarcinoma or squamous cell carcinoma.

It has been reported that HPV persistent infection is one of the risk factors for cervical cancer 2. The main virulence proteins of HPV are E6 protein and E7 protein. E6 protein can bind to ubiquitin transferase protein E6AP to degrade P53 and promote CDK2/Cyclin E complex to phosphorylate RB. E7 can interact with the CUL2 complex and lead to RB degradation. These processes ultimately led to the activation of transcription complexes E2F1 and DP-1, which induced cell S-phase progression 4. Interestingly, genes E6 and E7 are adjacent genes and co-transcripts. Therefore, when designing the experimental protocol, we can only select two proteins to knockdown simultaneously. Bioinformatics analysis revealed that the transcription factor E2F1 is likely to bind to the promoter region of MELK and enhance the transcription of MELK. Through the detection of protein levels and RNA levels, we did find a link between the three. At the same time, a recent melanoma study found that E2F1 is the upstream transcription factor of MELK 31. Despite the lack of some in-depth experimental evidence, combining some existing studies with our experimental data, we determined that E6 protein and E7 protein regulate the MELK gene by promoting the activity of the E2F1 and DP-1 transcription complexes.

For cervical cancer, its early clinical symptoms are not obvious. When apparent symptoms appear, it is often in the middle and late stages, which poses a challenge to treatment. Finding new treatment strategies and improving patient survival will be the top priority. Therefore, alternative methods to effectively treat cervical cancer need to be developed. It has been reported that seven patients (78%) had a high T-cell response to MELK peptide, which indicated that MELK might be the target of immunotherapy in cervical cancer patients 32. We found that MELK is a survival kinase for cervical cancer cells, and MELK inhibition blocked the growth of cervical cancer cells. *In vitro* and *in vivo* experiments have shown that MELK targeted specific shRNA has a significant inhibitory effect on the growth of cervical cancer cells. Hence, we studied the impact of pharmacological inhibition of MELK using MELK-8A 28. The new small-molecule inhibitor MELK-8A inhibited the kinase activity and caused polyploidy in tumor cells, accompanied by apoptosis. The cell nucleus of the experimental group became larger than that of the control group. This phenomenon appears to be similar to the anti-tumor drug microtubule inhibitor (BPROLO75). The anti-tumor drug works by inhibiting microtubule polymerization, arresting cells in the G2/M phase, and forming multinucleated cells, allowing tumors to initiate apoptosis 33.

Paul-like protein kinase (PLK1) is a serine/threonine-like protein kinase that plays an important role in cell mitosis by phosphorylating different substrates, including promoting centrosome maturation, mitotic initiation, chromosome segregation, cytosol separation, etc. 34. It has been well documented that Aurora A-mediated T210 phosphorylation (pT210) of PLK1 is critical for PLK1 kinase activity 35. Activated PLK1 is capable of phosphorylating the bispecific protein kinase CDC25 and lowering the level of the nuclear kinase Wee1 36 37, which plays a vital role in the entry, maintenance, and withdrawal of mitosis. It has also been confirmed that phosphorylation of Tyr217 of PLK1 inhibits the phosphorylation activity of Thr210, leading to the inactivation of PLK1 38. The CDK1 and cyclin B complexes regulate cell mitosis. During the interphase of the cell, the Tyr15 site of CDK1 is phosphorylated, rendering the complex inactive. The protein kinase Wee1 can up-regulate the phosphorylation of Tyr15 of CDK1, and the bispecific protein kinase CDC25C can promote the dephosphorylation of Tyr15 of CDK1 39. We determined that inhibition of MELK kinase activity up-regulated the phosphorylation level of PLK1 and CDC25C, the protein level of Wee1, which in turn up-regulated the Try15 phosphorylation of CDK1 and led to the inactivation of CDK1 and CyclinB complexes. Eventually, tumor cells underwent the G2/M phase arrest, which triggers apoptosis. However, it is unclear why the cells are polyploid and why the size of the nucleus increases when MELK-8A inhibits MELK kinase activity.

From the published data, we found that MELK in Hela cells was active in transcriptional activities and translation activities in the G2/M phase. To further reveal the role of MELK in the mitosis of Hela cervical cancer cells, we performed a bioinformatics analysis of the MELK interactome. The analysis found that the main interactors of MELK are closely related to the translation initiation and protein folding, such as ribosome component protein, tubulin protein family, and a certain number of apoptosis-related proteins or cell cycle regulatory related proteins, such as DYNLL1, SFN, YWHAE, YWHAZ, YWHAQ, etc. The function of these proteins suggests that MELK is involved in the translation and folding of proteins during mitosis and can directly regulate cell cycle progression and apoptosis by acting on related proteins.

However, some limitations should be noted. First, we used bioinformatics methods to predict transcription factors of MELK that may not be the only or main regulators. Our experimental results also suggest that MELK remains weakly expressed after the E2F1 knockdown. Second, due to the ease and convenience of the cell lines, we have used mainly HeLa cell lines (HPV 18 positive) in our study instead of human primary cells or other HPV-infected cell lines. This issue may make some experimental results different from clinical samples. Third, we do not have a comprehensive identification of the downstream phosphorylation substrates of MELK, although the major phosphorylation substrates should be present in the interactome. It has been shown that the phosphorylation of MELK has a strong preference for arginine at the -3 position relative to the phosphorylation site. Among our MELK interactome that fit this phosphorylation motif are MELK itself, RPL15, SQSTM1, etc. Among them, SQSTM1 has been reported to be phosphorylated by MELK in melanoma, which activates the NF-κB pathway and promotes cancer cell growth 40. The cascade reaction of these substrates should be the main downstream pathway of MELK and need more research. Inhibition of this pathway may be responsible for the polyploid cell generation and apoptosis that results from inhibition using MELK-8A.

In conclusion, we demonstrated for the first time that HPV 18 E6/E7 could regulate MELK through transcription factor E2F1 in cervical cancer. It is involved in protein translation and folding during cell division, and its absence or inactivation of expression will lead to abnormal mitosis and cell apoptosis. Our results underpin a vital role for MELK, and it may serve as a therapeutic target for cervical cancer.

## Supplementary Material

Supplementary figures.

Supplementary table 1.

Supplementary table 2.

Supplementary table 3.

## References

[1] Bray F, Ferlay J, Soerjomataram I, Siegel RL, Torre LA, Jemal A (2018). Global cancer statistics 2018: GLOBOCAN estimates of incidence and mortality worldwide for 36 cancers in 185 countries. CA Cancer J Clin.

[2] Crosbie EJ, Einstein MH, Franceschi S, Kitchener HC (2013). Human papillomavirus and cervical cancer. Lancet.

[3] Woodman CB, Collins SI, Young LS (2007). The natural history of cervical HPV infection: unresolved issues. Nat Rev Cancer.

[4] Hoppe-Seyler K, Bossler F, Braun JA, Herrmann AL, Hoppe-Seyler F (2018). The HPV E6/E7 Oncogenes: Key Factors for Viral Carcinogenesis and Therapeutic Targets. Trends Microbiol.

[5] Rajkumar T, Sabitha K, Vijayalakshmi N, Shirley S, Bose MV, Gopal G (2011). Identification and validation of genes involved in cervical tumourigenesis. BMC Cancer.

[6] Wang J, Wang Y, Shen F, Xu Y, Zhang Y, Zou X (2018). Maternal embryonic leucine zipper kinase: A novel biomarker and a potential therapeutic target of cervical cancer. Cancer Med.

[7] Moravcevic K, Mendrola JM, Schmitz KR, Wang YH, Slochower D, Janmey PA (2010). Kinase associated-1 domains drive MARK/PAR1 kinases to membrane targets by binding acidic phospholipids. Cell.

[8] Heyer BS, Warsowe J, Solter D, Knowles BB, Ackerman SL (1997). New member of the Snf1/AMPK kinase family, Melk, is expressed in the mouse egg and preimplantation embryo. Mol Reprod Dev.

[9] Blot J, Chartrain I, Roghi C, Philippe M, Tassan JP (2002). Cell cycle regulation of pEg3, a new Xenopus protein kinase of the KIN1/PAR-1/MARK family. Developmental biology.

[10] Ganguly R, Mohyeldin A, Thiel J, Kornblum HI, Beullens M, Nakano I (2015). MELK-a conserved kinase: functions, signaling, cancer, and controversy. Clin Transl Med.

[11] Marie SK, Okamoto OK, Uno M, Hasegawa AP, Oba-Shinjo SM, Cohen T (2008). Maternal embryonic leucine zipper kinase transcript abundance correlates with malignancy grade in human astrocytomas. Int J Cancer.

[12] Choi S, Ku JL (2011). Resistance of colorectal cancer cells to radiation and 5-FU is associated with MELK expression. Biochem Biophys Res Commun.

[13] Pickard MR, Green AR, Ellis IO, Caldas C, Hedge VL, Mourtada-Maarabouni M (2009). Dysregulated expression of Fau and MELK is associated with poor prognosis in breast cancer. Breast Cancer Res.

[14] Kuner R, Falth M, Pressinotti NC, Brase JC, Puig SB, Metzger J (2013). The maternal embryonic leucine zipper kinase (MELK) is upregulated in high-grade prostate cancer. J Mol Med (Berl).

[15] Badouel C, Chartrain I, Blot J, Tassan JP (2010). Maternal embryonic leucine zipper kinase is stabilized in mitosis by phosphorylation and is partially degraded upon mitotic exit. Experimental cell research.

[16] Wang Y, Li YM, Baitsch L, Huang A, Xiang Y, Tong H (2018). Correction: MELK is an oncogenic kinase essential for mitotic progression in basal-like breast cancer cells. Elife.

[17] Whittier KL, Boese EA, Gibson-Corley KN, Kirby PA, Darbro BW, Qian Q (2013). G-protein coupled receptor expression patterns delineate medulloblastoma subgroups. Acta neuropathologica communications.

[18] Tung MC, Hsieh SC, Yang SF, Cheng CW, Tsai RT, Wang SC (2013). Knockdown of lipocalin-2 suppresses the growth and invasion of prostate cancer cells. The Prostate.

[19] Fiebig HH, Berger DP, Winterhalter BR, Plowman J (1990). *In vitro* and *in vivo* evaluation of US-NCI compounds in human tumor xenografts. Cancer Treat Rev.

[20] Yu G, Wang LG, Han Y, He QY (2012). clusterProfiler: an R package for comparing biological themes among gene clusters. OMICS.

[21] Yu G, Li F, Qin Y, Bo X, Wu Y, Wang S (2010). GOSemSim: an R package for measuring semantic similarity among GO terms and gene products. Bioinformatics.

[22] Ito K, Murphy D (2013). Application of ggplot2 to Pharmacometric Graphics. CPT Pharmacometrics Syst Pharmacol.

[23] Gu Z, Eils R, Schlesner M (2016). Complex heatmaps reveal patterns and correlations in multidimensional genomic data. Bioinformatics.

[24] Goodwin EC, DiMaio D (2000). Repression of human papillomavirus oncogenes in HeLa cervical carcinoma cells causes the orderly reactivation of dormant tumor suppressor pathways. Proceedings of the National Academy of Sciences.

[25] Hiebert S, Chellappan S, Horowitz J, Nevins J (1992). The interaction of RB with E2F coincides with an inhibition of the transcriptional activity of E2F. Genes & development.

[26] Khleif SN, DeGregori J, Yee CL, Otterson GA, Kaye FJ, Nevins JR (1996). Inhibition of cyclin D-CDK4/CDK6 activity is associated with an E2F-mediated induction of cyclin kinase inhibitor activity. Proceedings of the National Academy of Sciences.

[27] Yim E-K, Park J-S (2005). The role of HPV E6 and E7 oncoproteins in HPV-associated cervical carcinogenesis. Cancer research and treatment.

[28] Toure BB, Giraldes J, Smith T, Sprague ER, Wang Y, Mathieu S (2016). Toward the Validation of Maternal Embryonic Leucine Zipper Kinase: Discovery, Optimization of Highly Potent and Selective Inhibitors, and Preliminary Biology Insight. Journal of medicinal chemistry.

[29] Franz M, Rodriguez H, Lopes C, Zuberi K, Montojo J, Bader GD (2018). GeneMANIA update 2018. Nucleic acids research.

[30] Shannon P, Markiel A, Ozier O, Baliga NS, Wang JT, Ramage D (2003). Cytoscape: a software environment for integrated models of biomolecular interaction networks. Genome research.

[31] Janostiak R, Rauniyar N, Lam TT, Ou J, Zhu LJ, Green MR (2017). MELK Promotes Melanoma Growth by Stimulating the NF-kappaB Pathway. Cell Rep.

[32] Hasegawa K, Ikeda Y, Kunugi Y, Kurosaki A, Imai Y, Kohyama S (2018). Phase I Study of Multiple Epitope Peptide Vaccination in Patients With Recurrent or Persistent Cervical Cancer. Journal of immunotherapy (Hagerstown, Md: 1997).

[33] Kuo CC, Hsieh HP, Pan WY, Chen CP, Liou JP, Lee SJ (2004). BPR0L075, a novel synthetic indole compound with antimitotic activity in human cancer cells, exerts effective antitumoral activity *in vivo*. Cancer research.

[34] de Carcer G, Manning G, Malumbres M (2011). From Plk1 to Plk5: functional evolution of polo-like kinases. Cell Cycle.

[35] Lowery DM, Lim D, Yaffe MB (2005). Structure and function of Polo-like kinases. Oncogene.

[36] Qian YW, Erikson E, Maller JL (1999). Mitotic effects of a constitutively active mutant of the Xenopus polo-like kinase Plx1. Mol Cell Biol.

[37] Owens L, Simanski S, Squire C, Smith A, Cartzendafner J, Cavett V (2010). Activation domain-dependent degradation of somatic Wee1 kinase. J Biol Chem.

[38] Caron D, Byrne DP, Thebault P, Soulet D, Landry CR, Eyers PA (2016). Mitotic phosphotyrosine network analysis reveals that tyrosine phosphorylation regulates Polo-like kinase 1 (PLK1). Science signaling.

[39] Takizawa CG, Morgan DO (2000). Control of mitosis by changes in the subcellular location of cyclin-B1-Cdk1 and Cdc25C. Current opinion in cell biology.

[40] Janostiak R, Rauniyar N, Lam TT, Ou J, Zhu LJ, Green MR (2017). MELK Promotes Melanoma Growth by Stimulating the NF-κB Pathway. Cell reports.

