# Supplementary material for: Up-regulation of MELK by E2F1 promotes the proliferation in cervical cancer cells
Source: Int J Biol Sci. 2021 Sep 7;17(14):3875–88. doi: 10.7150/ijbs.62517 (PMC8495384; doi:10.7150/ijbs.62517)
Supplement: Supplementary file 1 — Supplementary figures. [file ijbsv17p3875s1.pdf]

Figure S1

A Expression of MELK in CESC based on patient's race from TCGA-CESE project.

B Expression of MELK in CESC based on patient's weight from TCGA-CESE project.

C Expression of MELK in CESC based on patient's age (years) from TCGA-CESE project.

D Bioinformatics analysis indicates that E2F1 is likely to be the upstream transcription factor of MELK in cervical cancer. E2F1 peaks at the promoter of MELK (chr9:36,569,218-36,579,655).

Figure S2

A Viability of C33A cells 24 hours posttreatment with MELK-8A. Cell viability in a dose-dependent manner 24 hours posttreatment. DMSO was used as a control.

B Effect of MELK kinase activity on proliferation after 5μM MELK-8A inhibition. The cell viability of SiHa and C33A cells treated with MELK-8A was measured every 24 hours for 72 hours using the CCK8 cell proliferation assay kit. * $P < 0.05$, ** $P < 0.01$, *** $P < 0.001$ and **** $P < 0.0001$ compared to control.

Figure S1

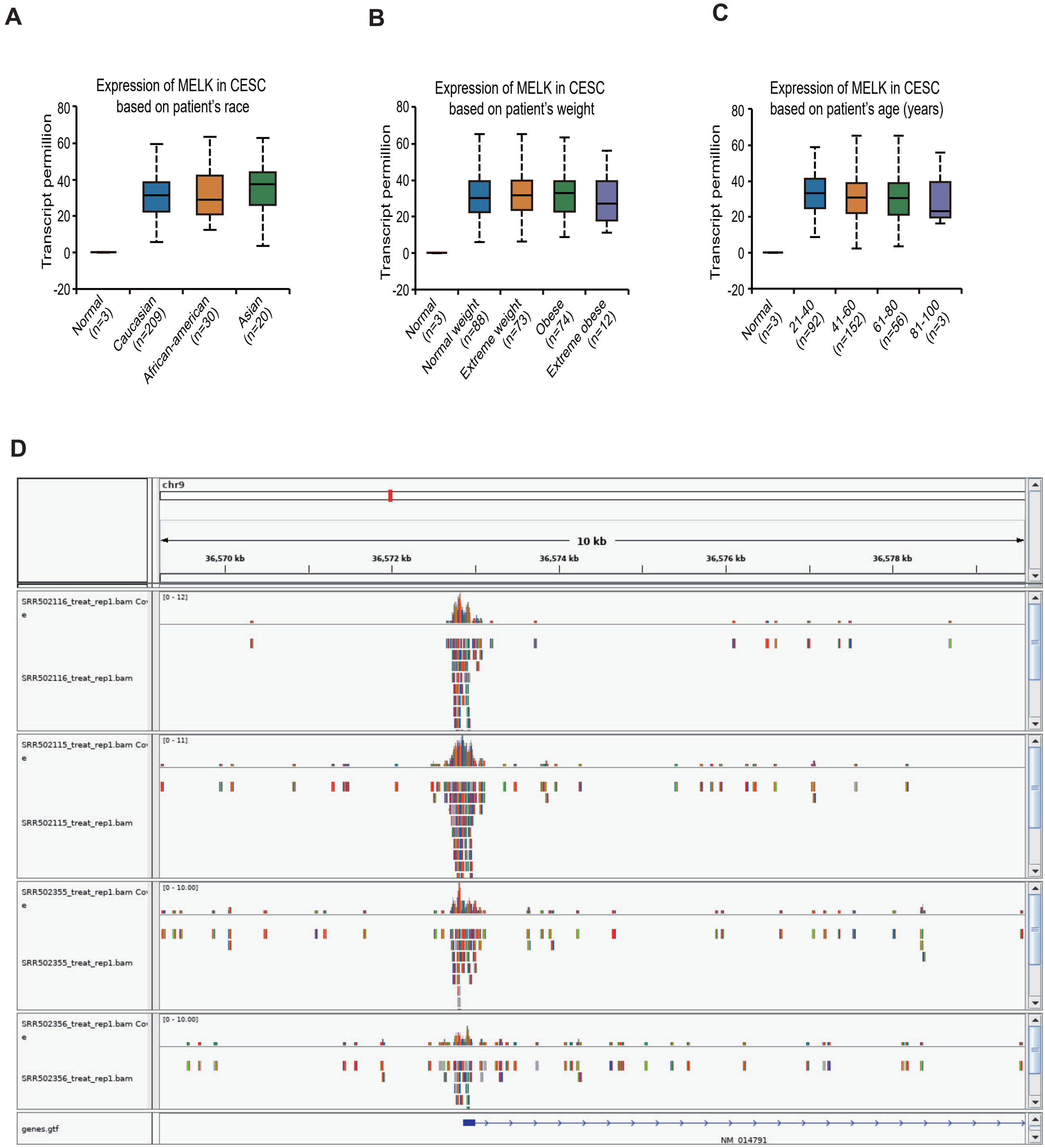
A
Expression of MELK in CESC
based on patient's race
Transcript permillion
80
60
40
20
0
-20
Normal (n=3)
Caucasian (n=209)
African-american (n=30)
Asian (n=20)
B
Expression of MELK in CESC
based on patient's weight
Transcript permillion
Normal (n=3)
Normal weight (n=88)
Extreme weight (n=73)
Obese (n=74)
Extreme obese (n=12)
C
Expression of MELK in CESC
based on patient's age (years)
Transcript permillion
Normal (n=3)
21-40 (n=92)
41-60 (n=152)
61-80 (n=56)
81-100 (n=3)
D
chr9
10 kb
36,570 kb
36,572 kb
36,574 kb
36,576 kb
36,578 kb
SRR502116_treat_rep1.bam Coverage
SRR502116_treat_rep1.bam
SRR502115_treat_rep1.bam Coverage
SRR502115_treat_rep1.bam
SRR502355_treat_rep1.bam Coverage
SRR502355_treat_rep1.bam
SRR502356_treat_rep1.bam Coverage
SRR502356_treat_rep1.bam
genes.gtf
NM_014791

Figure S2

A

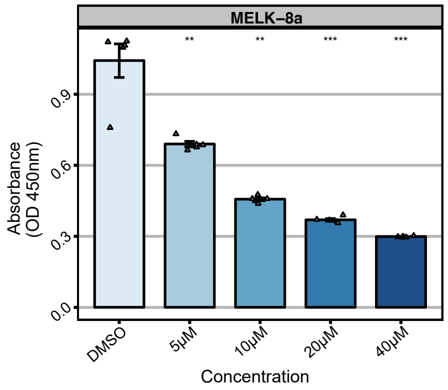
MELK–8a
Absorbance
(OD 450nm)
0.0
0.3
0.6
0.9
**
**
***
***
DMSO
5μM
10μM
20μM
40μM
Concentration

C

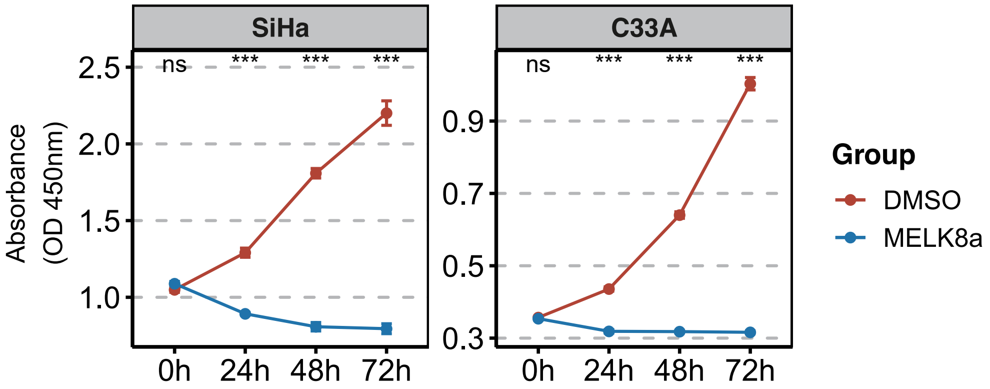
SiHa
C33A
Absorbance
(OD 450nm)
2.5
2.0
1.5
1.0
0.9
0.7
0.5
0.3
ns
***
***
***
0h
24h
48h
72h
Group
DMSO
MELK8a
